# Serum α-synuclein and IL-1β are increased and correlated with measures of disease severity in children with epilepsy: potential prognostic biomarkers?

**DOI:** 10.1186/s12883-020-01662-y

**Published:** 2020-03-09

**Authors:** Jieun Choi, Soo Yeon Kim, Hunmin Kim, Byung Chan Lim, Hee Hwang, Jong Hee Chae, Ki Joong Kim, Sohee Oh, Eun Young Kim, Jeon-Soo Shin

**Affiliations:** 1 Department of Pediatrics, Seoul National University College of Medicine, Seoul Metropolitan Government Seoul National University Boramae Medical Center, Boramaero 5 gil 20, Dongjakgu, Seoul, 07061 South Korea; 2 Department of Pediatrics, Pediatric Clinical Neuroscience Center, Seoul National University Children’s Hospital, Seoul National University College of Medicine, Seoul, South Korea; 3 Department of Pediatrics, Seoul National University Bundang Hospital, Seoul, South Korea; 4 Department of Biostatistics, SMG-SNU Boramae Medical Center, Seoul National University College of Medicine, Seoul, South Korea; 5 Department of Microbiology, Brain Korea 21 Plus Project for Medical Science, Severance Biomedical Science Institute and Institute for Immunology and Immunological Diseases, Yonsei University College of Medicine, 50-1 Yonsei-ro Seodaemoon-gu Seoul, Seoul, 03722 South Korea

**Keywords:** α-Synuclein, IL-1β, Epilepsy, Children, Acquired demyelinating disorders

## Abstract

**Background:**

The search for noninvasive biomarkers of neuroinflammation and neurodegeneration has focused on various neurological disorders, including epilepsy. We sought to determine whether α-synuclein and cytokines are correlated with the degree of neuroinflammation and/or neurodegeneration in children with epilepsy and with acquired demyelinating disorders of the central nervous system (CNS), as a prototype of autoimmune neuroinflammatory disorders.

**Methods:**

We analyzed serum and exosome levels of α-synuclein and serum proinflammatory and anti-inflammatory cytokines among 115 children with epilepsy and 10 acquired demyelinating disorders of the CNS and compared to 146 controls. Patients were enrolled prospectively and blood was obtained from patients within 48 h after acute afebrile seizure attacks or relapse of neurological symptoms. Acquired demyelinating disorders of the CNS include acute disseminated encephalomyelitis, multiple sclerosis, neuromyelitis optica spectrum disorders, and transverse myelitis. The controls were healthy age-matched children. The serum exosomes were extracted with ExoQuick exosome precipitation solution. Serum α-synuclein levels and serum levels of cytokines including IFN-β, IFN-γ, IL-1β, IL-6, IL-10 and TNF-α were measured using single and multiplex ELISA kits. Data were analyzed and compared with measures of disease severity, such as age at disease onset, duration of disease, and numbers of antiepileptic drug in use.

**Results:**

Serum α-synuclein levels were significantly increased in patients with epilepsy and acquired demyelinating disorders of the CNS compared to controls (both, *p < 0.*05) and showed correlation with measures of disease severity both in epilepsy (*p* < 0.05, *r* = 0.2132) and in acquired demyelinating disorders of the CNS (*p* < 0.05, *r* = 0.5892). Exosome α-synuclein showed a significant correlation with serum α-synuclein (*p* < 0.0001, *r* = 0.5915). Serum IL-1β levels were correlated only with the numbers of antiepileptic drug used in children with epilepsy (*p* < 0.001, r = 0.3428), suggesting drug resistant epilepsy.

**Conclusions:**

This is the first study in children demonstrating that serum α-synuclein levels were significantly increased in children with epilepsy and with acquired demyelinating disorders of the CNS and correlated with measures of disease severity. Serum IL-1β levels showed significant correlation only with drug resistance in children with epilepsy. Thus, these data support that serum levels of α-synuclein and IL-1β are potential prognostic biomarkers for disease severity in children with epilepsy.

CNS, central nervous system.

## Background

Pediatric epilepsy is a chronic brain disorder accompanied by behavioral and cognitive problems, which include intellectual dysfunctions and attention deficits. The International League Against Epilepsy (ILAE) defines epilepsy as a condition in which a patient has “two or more unprovoked seizure attacks occurring at least 1 day apart” [1]. Approximately one-third of patients with epilepsy have drug resistant epilepsy, defined as epilepsy that is not controlled by two antiepileptic medications to sustain seizure freedom [2]. Some children with drug resistant epilepsy may show regression in intellectual abilities over time [3]. The causes of regression may include the etiology of the underlying epilepsy, the spike discharges on electroencephalogram (EEG) outputs, the seizures themselves and a variety of medications [4, 5], but there is no known biomarker for disease severity or neurocognitive comorbidity.

α-Synuclein is one of the most abundant proteins within the neurons of the brain [6–8]. This protein is suggested to participate in synaptic transmission by regulating neurotransmitter release and vesicle recycling, also in mitochondrial and synaptic dysfunction and neuronal apoptosis and in Ca^2+^ homeostasis [9, 10]. Oligomeric forms of α-synuclein have been demonstrated to increase synaptic transmission and to decrease long-term potentiation and certain oligomeric forms were reported to play a key role in the neurodegenerative process [11]. Intracellular deposition of α-synuclein were found in several neurodegenerative diseases presenting with cognitive problems and dementia [12].

α-Synuclein levels have been investigated in patients with various neurological disorders. Soluble oligomeric α-synuclein has been suggested as a potential biomarker for Parkinson’s disease because of its increased plasma levels in patients [13]. Exosome α-synuclein is mostly specific to the CNS and is found increased in patients with Parkinson’s disease [14]. Cerebrospinal fluid (CSF) α-synuclein levels are reported to be elevated in patients with multiple sclerosis and neuromyelitis optica during relapse [15] as well as in traumatic brain injury [16]. Multiple sclerosis and neuromyelitis optica are the main autoimmune inflammatory demyelinating disorders of the CNS during childhood. Chronic autoimmune neuroinflammation is the most important component of the pathology of these diseases and the progressive clinical disability, such as motor dysfunction and cognitive declines [17].

Clinical evidences have shown that neuroinflammation is a hallmark of the epileptic focus in drug-resistant epilepsy [18, 19]. Neuroinflammatory mechanisms contribute to seizure generation in animal models of epilepsy [19]. Substantial effort has been invested in the search for noninvasive biomarkers of neuroinflammation; this search has focused on various neurological disorders including autoimmune neurological diseases, neurocognitive neurodegenerative diseases and neuropsychiatric dysfunction as well as epilepsy [20]. CSF/serum IL-1β levels have been reported as a prognostic factor for epilepsy development after traumatic brain injury [21], and the brain-specific adhesion molecule ICAM5 can be used to discriminate between drug-responsive and drug-resistant epilepsy [22]. In medial temporal lobe epilepsy, α-synuclein deposition has been found in the hippocampus along with neuronal cell loss and reactive gliosis [23]. Adult patients with intractable epilepsy showed elevated α-synuclein levels in both CSF and serum [24]. However, there are no reports about α-synuclein levels in children with epilepsy, who usually have earlier disease onset and are more likely to develop intellectual disability and behavioral problems.

We aimed to determine whether serum and exosomal levels of α-synuclein and cytokines are correlated with degrees of neuroinflammation and neurodegeneration in children with epilepsy and a prototypical class of neuroinflammatory disorders, namely, acquired demyelinating disorders of the CNS. For this purpose, we analyzed serum and exosomal levels of α-synuclein and serum proinflammatory and anti-inflammatory cytokines among children with epilepsy and acquired demyelinating disorders of the CNS and compared to controls.

## Methods

### Patient information

We prospectively enrolled 115 epilepsy patients having afebrile seizure attacks within the last 48 h before visiting the pediatric neurology clinic of Seoul National University Boramae Medical Center. Epilepsy was diagnosed and classified according to the criteria established in the 2017 International Classification of Epileptic Syndromes [25]. Children with epilepsy underwent examinations that included medical history, physical and neurological examinations, electroencephalograms, blood tests (including metabolic and autoimmune studies) and brain magnetic resonance imaging (MRI). Autoimmune epilepsy, neonatal seizures, infantile spasms, posttraumatic epilepsy and post-encephalitic epilepsy were excluded. Ten patients with acquired demyelinating disorders of the CNS were included and diagnosed according to the revised definitions proposed by the International Pediatric Multiple Sclerosis Study Group [26]. Acquired demyelinating disorders of the CNS include acute disseminated encephalomyelitis (ADEM), multiple sclerosis (MS), neuromyelitis optica spectrum disorder (NMOSD), and transverse myelitis (TM) [26]. MS were diagnosed based on the criteria proposed in 2010 by McDonald [27] and NMOSD were diagnosed based on the 2015 Wingerchuk diagnostic criteria [28]. TM were diagnosed based on the 2013 International Pediatric Multiple Sclerosis Study Group criteria for TM [26]. Among ADEM, MS, NMO and TM, blood tests were performed two to five times in a patient at the time of relapse or worsening of symptoms to measure differences from the initial attack and relapse; each test was counted as separate and compared serially with the disease duration. So, total numbers of tests were 18 times, and we analyzed each tests individually. For correlation analysis of serum α-synuclein and cytokine levels with measures of disease severity, we quantified disease severity using the age of disease onset, the duration of disease, the duration of the last seizure before the blood collection and the number of antiepileptic medications in patients with epilepsy and the age of disease onset and the duration of disease in patients with acquired demyelinating disorders of the CNS. The 146 controls were healthy age-matched children who visited headache clinics for routine workup but did not have acute current headache attacks or any history of febrile seizures, epilepsy, or acquired demyelinating disorders of the CNS. This study was approved by the Institutional Review Board at the Seoul National University Boramae Medical Center. Informed written consent was obtained from the parent of each child, and verbal assent was obtained from each child or adolescent.

### Serum and exosome purification

Blood was obtained from patients within 48 h after acute seizure attacks or relapse of neurologic symptoms such as visual loss or sudden motor deficits. Serum was separated and frozen for subsequent cytokine and α-synuclein assays. Control blood serum was collected and frozen as above.

The serum exosomes were extracted with ExoQuick exosome precipitation solution (System Biosciences, CA, USA) [29]. Briefly, the serum was centrifuged at 3000 *g* at 4 °C for 15 min. Then, the supernatant and one-fourth of its volume in ExoQuick Solution were mixed well and incubated at 4 °C. After 2 h, the mixture was centrifuged for 30 min at 1500 *g*, after which the pellet was dissolved and re-centrifuged for 5 min at 4 °C. The exosome-containing pellet was re-suspended in nuclease-free water.

### α-Synuclein and cytokine measurement

Serum α-synuclein levels were measured using commercially available enzyme-linked immunosorbent assay (ELISA) kits (Thermo Scientific, Waltham, MA [24]). Serum levels of cytokines including interferon (IFN)-β, IFN-γ, interleukin (IL)-1β, IL-6, IL-10 and tumor necrosis factor (TNF)-α were measured using commercially available multiplex kits (Merck, Darmstadt, Germany) according to the manufacturer’s instructions. Samples were analyzed in duplicate and compared with controls. The lower limits of detection were 0.2 ng/mL for α-synuclein, 115.1 pg/ml for IFN-β, 0.8 pg/ml IFN-γ, 0.8 pg/mL for IL-1β, 0.9 pg/mL for IL-6, 1.1 pg/mL for IL-10 and 0.7 pg/mL for TNF-α.

### Statistical analysis

The data are presented as the mean ± 1 standard deviation (SD) (α-synuclein and cytokine levels). The Mann-Whitney test was used to compare the serum levels of α-synuclein and cytokines in patients with epilepsy and acute demyelinating disorders of the CNS versus controls. The Kruskal-Wallis test was used to compare serum or exosomal levels of α-synuclein between all patients and controls. Spearman’s rank correlation coefficient was calculated to detect significant correlations of α-synuclein levels and other cytokines or clinical variables with disease severity in the patient groups. GraphPad Prism v. 7.0 for Windows version 7.03 (GraphPad Software Inc., San Diego, CA, USA) was used to perform the above tests. Values are expressed as the means, and the threshold for statistical significance was set as *p* < 0.05 for all tests.

## Results

### Patient characteristics

The demographic and clinical features of the patients are shown in Table 1. One hundreds fifteen epilepsy patients, 10 patients with acquired demyelinating disorders of the CNS and 146 control children were included in this study. Numbers of tests done in children with acquired demyelinating disorders of the CNS were counted as 18 because several tests were done in same patients during relapse of neurological symptoms. Then mean age at time of tests were 8.9 years old in patients with epilepsy and 8.1 years old in patients with acquired demyelinating disorders of the CNS. The mean age at the time of tests were 9.4 years old in controls and showed no significant statistical differences. Mean duration of diseases were 3.1 years in epilepsy and 2.1 years in acquired demyelinating disorders of the CNS. Types and etiologies in the epilepsy patients are shown in Table 2. Etiologies of epilepsy were 3% structural, 10% genetic and 87% unknown. Duration of epilepsy were less than 1 year in 55% of patients, and 34% of patients had more than 3 years. The mean duration of the last seizure before the blood collection were 8.2 min and 72% were less than 5 min. 60% of patients had been controlled epilepsy under 1 anti-epileptic medications but 25% of patients had taken more than 3 anti-epileptic medications to control seizures. Patients with acquired demyelinating disorders of the CNS include 2 with ADEM, 1 with relapsing–remitting MS, 3 with NMO and 2 with TM (Table 3). One MS, 2 NMO and 1 TM patient were tested two to five times when their neurological symptoms were aggravated or relapsing; therefore, the total number of tests was counted as 18 (Table 1 and Table 3). Repeated tests were performed in one MS (3 times), 2 NMOSD (2 times and 5 times) and 1 TM (3 times) at the time of relapse or worsening of symptoms (Table 3).

**Table 1:** Demographic characteristics of the patients

	Control	Epilepsy	Acquired demyelinating disorders of the CNS
Number of subjects	146	115	10
Number of tests	146	115	18
Male/Female	76/68	68/47	3/7
Age at test (years; mean ± SD)	9.4 ± 2.9	8.9 ± 6.2	8.1 ± 4.3
Age at 1st symptom onset (years; mean ± SD)	NA	5.8 ± 5.2	6.1 ± 4.0
Duration of disease (year; mean ± SD)	NA	3.1 ± 4.5	2.1 ± 2.9

CNS, central nervous system; SD, standard deviation; Inflammatory brain diseases include acute cerebellar ataxia, acute disseminated encephalomyelitis, multiple sclerosis, neuromyelitis optica spectrum disorder (*N* = 8) and transverse myelitis

**Table 2:** Characteristics of children with epilepsy

Epilepsy syndromes	% (N)	Duration of epilepsy (years)	% (N)
Benign epilepsy with centrotemporal spikes	16 (18)	< 1	55 (63)
Benign infantile epilepsy	2 (3)	1~3	13 (15)
Early myoclonic encephalopathy	1 (1)	3~5	8 (9)
Late onset childhood occipital epilepsy	2 (3)	≥ 5	24 (28)
Temporal lobe epilepsy	11 (12)		
Panayiotopoulos syndrome	4 (4)		
Generalized epilepsy with febrile seizure plus	7 (8)	**Age at seizure onset (years)**	% (N)
Childhood absence epilepsy	7 (8)	< 1	34 (39)
Juvenile myoclonic epilepsy	5 (6)	1~3	17 (19)
Lennox Gastaut syndrome	24 (28)	3~5	10 (12)
Idiopathic generalized epilepsy	16 (18)	5~7	12 (14)
Symptomatic generalized epilepsy	5 (6)	≥ 7	27 (31)
**Epilepsy etiology**	% (N)	**Duration of last seizure before blood collection (minutes)**	% (N)
Structural	3 (4)	< 5	83 (72)
Genetic	10 (11)	5~10	11 (10)
Dravet syndrome	5	10~30	7 (6)
Lafora diseases	3	≥ 30	14 (12)
Rett syndrome	2		
Infectious	0		
Immune	0		
**Neurodevelopmental comorbidities**	% (N)	**Number of antiepileptic drugs (N)**	% (N)
ADHD	18 (16)	1	60 (70)
Autism spectrum disorder	4 (3)	2	15 (17)
Mental retardation	31 (27)	≥ 3	25 (28)

N, number; ADHD, attention deficit hyperactivity disorder

**Table 3:** Characteristics of acquired demyelinating disorders of the central nervous system in children

Diagnosis	Age at test (years; mean ± SD)	Serum α-synuclein (ng/ml) (mean ± SD)	Patient ID	Number of tests	Number of relapses	Medications	Outcome
Acute disseminated encephalomyelitis	7.9 ± 1.8	9.5 ± 1.9	A	1	None	IVIG, PD	Completely recovered
			B	1	None	IVIG, PD	Completely recovered
Multiple sclerosis	13.9 ± 0.1	11.0 ± 7.4	C	3 times	3 times	IVIG+PD+ interferon-β	Relapsing–remitting type
Neuromyelitis optica spectrum disorder	8.7 ± 3.6	13.7 ± 4.8	D	1	2 times	IVIG+PD+ AZP + plasmapheresis	Expired
			E	2 times	3 times	IVIG+PD+ AZP	Rapid disease progression; bedridden with home ventilator
			F	5 times	4 times	IVIG+PD+ AZP	Slow disease progression, but can walk independently
Transverse myelitis	3.8 ± 1.8	10.8 ± 7.9	G	1	None	IVIG+PD	Rapidly and completely recovered
			H	1	None	IVIG+PD	Rapidly and completely recovered
			I	3 times	None	IVIG+PD	Unresponsive to treatment for the first two months, but slowly recovered

SD; standard deviation, ID; identification, IVIG, intravenous immunoglobulin; PD; prednisolone, AZP; azathioprine

#### Serum α-synuclein was significantly increased in patients with epilepsy and acquired demyelinating disorders of the CNS and was correlated with measures of disease severity

**Fig. 1:**
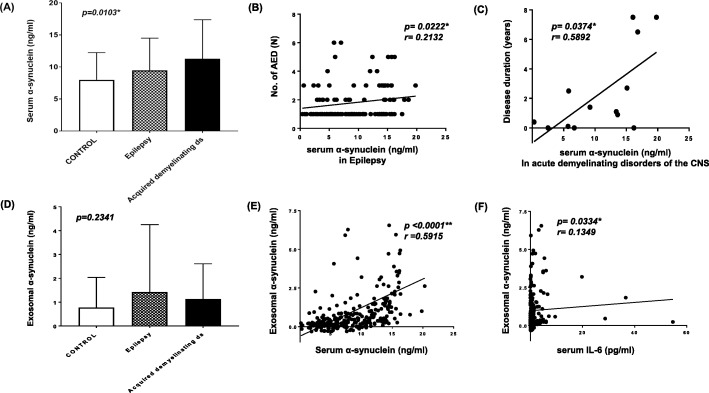
Correlation of serum and exosome α-synuclein with measures of disease severity in epilepsy and acquired demyelinating disorders of CNS. (**a**-**b**) Mean α-synuclein levels from serum (**a**) and exosomal extracts (**b**) in patients with epilepsy (*N* = 115), acquired demyelinating disorders of the central nervous system (*N* = 18) and controls (*N* = 146). Acquired demyelinating disorders of the central nervous system include acute disseminated encephalomyelitis, neuromyelitis optica spectrum disorder, multiple sclerosis and transverse myelitis. (**a**) Mean serum α-synuclein levels showed significant differences in patients with epilepsy and acquired demyelinating disorders of the CNS compared to controls (*p* = 0.0103). (**b**) Serum α-synuclein levels showed a significant positive correlation with the number of antiepileptic drugs used by epilepsy patients (*p* = 0.0222, *r* = 0.2132). (**c**) Serum α-synuclein levels showed a significant positive correlation with the duration of disease in patients with acute demyelinating disorders of the CNS (*p* = 0.0374, *r* = 0.5892). (**d**) Mean exosomal α-synuclein levels were not significantly different in patients with epilepsy or acquired demyelinating disorders of the central nervous system compared to controls (*p* = 0.2341). (**e**-**f**) Correlation of exosome α-synuclein levels with serum α-synuclein levels (**e**) and with serum IL-6 levels (**f**) in combined groups of patients with epilepsy (N = 115) plus patients with acquired demyelinating disorders of the CNS (N = 18) and controls (N = 146). (**e**) Exosome α-synuclein levels showed a significant correlation with serum α-synuclein positively (*p* < 0.0001, *r* = 0.5915). (**f**) Exosomal α-synuclein levels showed significant correlation with serum IL-6 levels positively (*p* = 0.0334, *r* = 0.1349). Ds, disorders; No, numbers; AED, antiepileptic drugs; CNS, central nervous system; IL, interleukin

Serum α-synuclein levels were significantly increased in patients with epilepsy and acquired demyelinating disorders of the CNS compared to controls (Tables 4, 9.5 and 11.2 vs 8.0 ng/ml, both *p* < 0.05). Serum α-synuclein levels were significantly different in patients with epilepsy and acquired demyelinating disorders of the CNS compared to controls, in three group comparisons (Fig. 1 (A), *p* < 0.05). In the subgroup of acquired demyelinating disorders of the CNS, serum α-synuclein was 13.7 ng/ml in NMO, 11.0 ng/ml in MS, 10.8 ng/ml in TM and 9.5 ng/ml in ADEM compared to 8.0 ng/ml in controls (Table 3).

**Table 4:** Comparisons of serum α-synuclein and cytokines between epilepsy, acquired demyelinating disorders of CNS and controls

Serum levels	Control (N = 146)	Epilepsy (N = 115)	*p*-value	Acquired demyelinating ds (N = 18)	*p*-value
α-Synuclein (ng/ml)	8.0 ± 4.3	9.5 ± 5.0	0.0160*	11.2 ± .5.9	0.0250*
IFN-β (pg/ml)	397.5 ± 312.3	475.5 ± 333.8	0.0646	814.9 ± 1227.3	0.4672
IFN-γ (pg/ml)	3.5 ± 9.3	4.6 ± 13.1	0.1718	6.1 ± 18.5	0.5644
IL-1β (pg/ml)	0.5 ± 1.0	3.5 ± 13.9	0.1038	0.4 ± 0.7	0.3141
IL-6 (pg/ml)	0.9 ± 1.4	4.2 ± 11.8	0.0052*	7.0 ± 15.5	0.1833
IL-10 (pg/ml)	4.0 ± 9.9	9.2 ± 20.9	< 0.0001**	17.7 ± 34.9	0.0120*
TNF-α (pg/ml)	10.2 ± 6.3	14.3 ± 14.8	0.0475*	12.1 ± 11.6	0.3959

Data, mean ± standard deviation; CNS, central nervous system; Acquired demyelinating ds, acquired demyelinating disorders of the central nervous system; IFN, interferon; IL, interleukin; TNF, tumor necrosis factor; *p < 0.05, **p < 0.001

Correlation analysis between serum α-synuclein and disease severity revealed that the number of antiepileptic medications and the duration of last seizure before the blood collection were correlated with serum α-synuclein and IL-6 levels in patients with epilepsy (Table 5 and Fig. 1 (B) & 3 (C); *p* < 0.05, *r* = 0.2132 and *p* < 0.05, *r* = 0.1884). In patients with acquired demyelinating disorders of the CNS, the duration of diseases was positively correlated with serum α-synuclein levels (Table 5 and Fig. 1 (C), *p* < 0.05, *r* = 0.5892). However, the age of disease onset did not correlate with serum α-synuclein levels in patients with epilepsy (Table 5, *p* = 0.6352) or with acquired demyelinating disorders of the CNS (Table 5, *p* = 0.4864).

**Table 5:** Correlations of serum α-synuclein and cytokines with measures of disease severity in epilepsy and acquired demyelinating disorders of CNS

Serum levels	Epilepsy (N = 115)								Acquired demyelinating ds (N = 18)			
	Age of disease onset		Duration of last sz before the blood collection		Number of AED		Disease duration		Age of disease onset		Disease duration	
	*p*-value	r	*p*-value	r	*p*-value	r	*p*-value	r	*p*-value	r	*p*-value	r
α-Synuclein (ng/ml)	0.6352		0.0438*	0.1884	0.0222*	0.2132	0.5575		0.4864		0.0374*	0.5892
IFN-β (pg/ml)	0.6274		0.8148		0.6119		0.6568		0.2188		0.3941	
IFN-γ (pg/ml)	< 0.0001**	−0.4197	0.4590		0.0235*	0.2288	0.0872		0.8242		0.6411	
IL-1β (pg/ml)	0.0110*	−0.2545	0.4913		0.0005**	0.3428	0.1359		0.8999		0.8044	
IL-6 (pg/ml)	0.0066*	−0.2714	0.0057*	0.2773	0.7776		0.3343		0.0429*	−0.6272	0.3101	
IL-10 (pg/ml)	0.0041*	−0.2864	0.6248		0.3572		0.7683		0.2273		0.4465	
TNF-α (pg/ml)	0.4018		0.7054		0.2542		0.6594		0.3781		0.3813	
Exosomal α-synuclein (ng/ml)	0.1042		0.1136		0.3488		0.2914		0.5298		0.0571	

CNS, central nervous system; Acquired demyelinating ds, acquired demyelinating disorders of the central nervous system; sz, seizure; AED, antiepileptic drugs; IFN, interferon; IL, interleukin; TNF, tumor necrosis factor; *p < 0.05, **p < 0.001

Serum α-synuclein did not showed significant correlation with serum levels of IFN-β, IFN-γ, IL-1β, IL-6, IL-10 and TNF-α in patients with epilepsy and acquired demyelinating disorders of the CNS.

#### Exosomal α-synuclein correlated with serum α-synuclein and serum IL-6 levels in patients with epilepsy and acquired demyelinating disorders of the CNS

Exosomal α-synuclein levels are increased in patients with epilepsy and acquired demyelinating disorders of the CNS compared with controls, although the difference was not statistically significant (Fig. 1 (D), *p* = 0.2341). Exosomal α-synuclein levels were higher in epilepsy and acquired demyelinating disorders of the CNS compared to controls (1.4 and 1.1 vs 0.8 ng/ml, *p* = 0.3828 and *p* = 0.1168), although neither difference was statistically significant. Exosomal levels of α-synuclein showed a significant correlation with serum levels of α-synuclein in patients with epilepsy and acquired demyelinating disorders of the CNS (Fig. 1 (E), *p* < 0.0001, *r* = 0.5915). Additionally, exosomal α-synuclein showed correlation with serum IL-6 levels in patients with epilepsy and acquired demyelinating disorders of the CNS (Fig. 1 (E), *p <* 0.05, *r* = 0.1349). However, exosomal α-synuclein levels did not show a significant correlation with the measures of disease severity, such as number of antiepileptic medication and duration of disease, both in patients with epilepsy and with acquired demyelinating diseases of the CNS (Table 5).

#### Serum cytokine profiles and correlation with the measures of disease severity

##### Negative correlation of serum levels of IFN-r, IL-1β, IL-6 and IL-10 with the age of disease onset in pediatric epilepsy patients with seizure attack within 48 h

Serum levels of proinflammatory cytokines, IL-6 and TNF-α were significantly increased in pediatric epilepsy patients with acute afebrile seizure attacks within 48 h compared to controls (Tables 4, 4.2 vs 0.9 pg/ml for IL-6, *p* < 0.05; 14.3 vs 10.2 pg/ml for TNF-α, *p* < 0.05). The anti-inflammatory cytokine IL-10 was also significantly increased compared to the controls (Tables 4, 9.2 vs 4.0 pg/ml, *p* < 0.0001). Serum levels of IFN-β, IFN-γ and IL-1β were higher compared to controls, although the differences were not statistically significant (Tables 4, 475.5 vs 397.5 for IFN-β, *p* = 0.0646; 4.6 vs 3.5 for IFN-γ, *p* = 0.178; 3.5 vs 0.5 pg/ml for IL-1β, *p* = 0.1038).

**Fig. 2:**
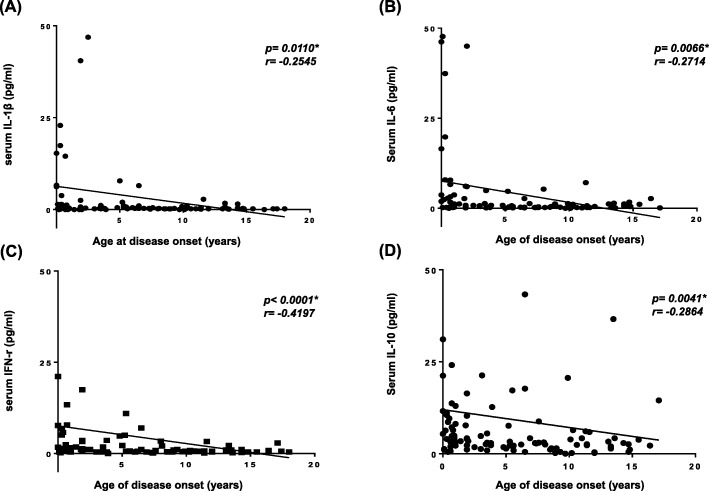
Correlation of serum levels of cytokines with age of disease onset in patients with epilepsy. (**a**-**d**) Correlation of serum levels of IL-1β (**a**), IL-6 (**b**), IFN-γ (**c**) and IL-10 (**d**) with age of disease onset in patients with epilepsy (N = 115). (**a**) Serum levels of IL-1β showed a significant negative correlation with age of disease onset in children with epilepsy (*p* = 0.0110, *r* = − 0.2545). (**b**) Serum levels of IL-6 showed a significant negative correlation with age of disease onset in children with epilepsy (*p* = 0.0066, *r* = − 0.2714). (**c**) Serum levels of IFN-γ showed a significant negative correlation with age of disease onset in children with epilepsy (*p* < 0.0001, *r* = − 0.4197). (**d**) Serum levels of IL-10 showed a significant negative correlation with age of disease onset in children with epilepsy (*p* = 0.0041, *r* = − 0.2864). IL, interleukin; IFN, interferon

Correlation analysis between serum cytokine levels and disease severity revealed that age at disease onset showed significant correlation negatively with serum levels of IFN-r, IL-1β, IL-6 and IL-10, indicating that the younger the patients were at disease onset, the more higher their cytokine levels to acute symptom of seizures (Table 5 and Fig. 2 (A-D), *p* < 0.0001, r = − 0.4197 for IFN-γ, *p* = 00110, r = − 0.2545 for IL-1β; *p* = 0.0066, r = − 0.2714 for IL-6; *p* = 0.0041, r = − 0.2864 for IL-10).

##### Positive correlation of serum levels of IL-1β and IFN-γ with numbers of antiepileptic medication and IL-6 with duration of last seizure before the blood collection in pediatric epilepsy patients with seizure attack within 48 h

**Fig. 3:**
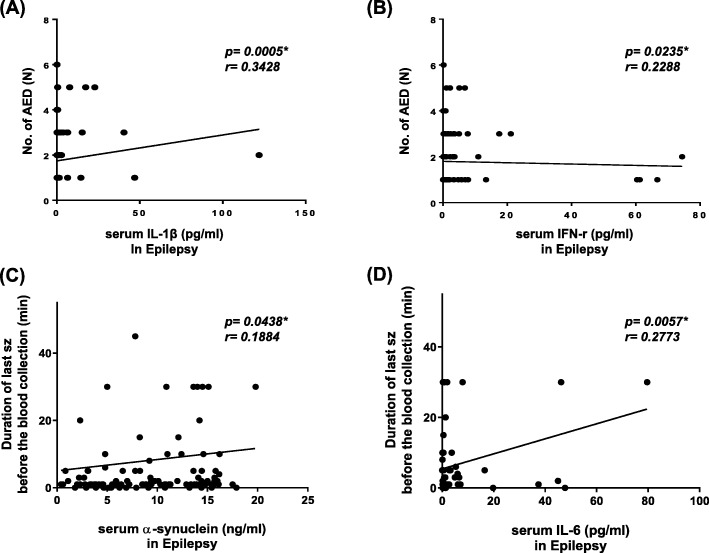
Correlation of serum α-synuclein with measures of the disease severity in epilepsy and acquired demyelinating disorders of CNS. (**a**-**b**) Correlation between the numbers of antiepileptic drug and serum levels of IL-1β (**a**) and IFN-γ (**b**). (**a**) Serum IL-1β levels showed a significant positive correlation with the numbers of antiepileptic drug taken for control of seizures in children with epilepsy (*p* = 0.0005, *r* = 0.3428). (**b**) Serum IFN-r levels showed a significant positive correlation with the numbers of antiepileptic drug for control of seizures in children with epilepsy (*p* = 0.0235, *r* = 0.2288) (**c**-**d**) Correlation between the duration of last seizure before the blood collection and serum levels of α-synuclein (**c**) and IL-6 (**d**). (**a**) Serum α-synuclein levels showed a significant positive correlation with the duration of last seizure before the blood collection in children with epilepsy (*p* = 0.0438, *r* = 0.1884). (**b**) Serum IL-6 levels showed a significant positive correlation with the duration of last seizure before the blood collection in children with epilepsy (*p* = 0.0057, *r* = 0.2773). CNS, central nervous system; No, numbers; AED, numbers of anti-epileptic drug; IL, interleukin; IFN, interferon; sz, seizure.

The numbers of antiepileptic drug showed significant correlation with serum levels of IL-1β and IFN-γ, positively (Table 5; Fig. 3 (A), *p* < 0.001, r = 0.3428 for IL-1β; Fig. 3 (B), *p* < 0.05, r = 0.2288 for IFN-γ). Disease durations in patients with epilepsy showed no significant correlation with serum levels of cytokines (Table 5). The duration of last seizure before the blood collection showed significant correlation with serum levels of IL-6, positively (Table 5; Fig. 3 (D), *p* < 0.05, r = 0.2773).

##### Increased serum IL-10 levels and negative correlation of serum IL-6 levels with age of disease onset in patients with acquired demyelinating disorders of the CNS

The serum IL-10 level was significantly increased in patients with acquired demyelinating disorders of the CNS compared to controls (Tables 4, 17.7 vs 4.0 pg/ml, *p* < 0.05). Serum levels of IFN-β, IFN-γ, and IL-6 were increased compared to controls, although the difference was not statistically significant (Tables 4, 814.9 vs 397.5 for IFN-β, 6.1 vs 3.5 for IFN-r, 7.0 vs 0.9 for IL-6). Other proinflammatory cytokines, including IL-1β and TNF-α, did not differ from the controls (Table 4).

Correlation analysis between serum cytokine levels and disease severity revealed that the age of disease onset was significantly correlated with serum levels of IL-6, which indicates that the younger the more aggravated cytokine responses to acute symptoms or relapse (Table 5, *p* = 0.0429, r = − 0.6272). The disease durations did not show significant correlation with serum levels of cytokines in patients with acquired demyelinating disorders of the CNS (Table 5).

## Discussion

This is the first study demonstrating that serum α-synuclein levels were significantly increased in children with epilepsy and acquired demyelinating disorders of the CNS and correlated with the measures of disease severity, suggesting neurodegenerative process. Exosome α-synuclein levels, probably CNS-derived, are correlated with serum levels of α-synuclein and IL-6. Serum levels of the proinflammatory cytokine IL-1β showed significant correlation with the measures of disease severity only in children with epilepsy but not in acquired demyelinating disorders in the CNS, implicating IL-1β as a potential prognostic biomarker for childhood epilepsy as ongoing neuroinflammation. Serum levels of IFN-γ, IL-1β, IL-6 and IL-10 showed negative correlation with age of disease onset in pediatric epilepsy patients, suggesting that younger patients demonstrated higher inflammatory responses to afebrile seizure attacks. Thus, these data suggest that serum levels of IL-1β are potential prognostic biomarker specific for children with epilepsy and serum levels of α-synuclein are a potential prognostic biomarker for both drug resistant epilepsy and acquired demyelinating disorders of the CNS in children.

Many molecules and pathways are activated after acute brain injuries, such as cerebrovascular damage, infections, seizures and traumatic brain injury, often leading to the development of chronic neuroinflammation not only in typical neuroinflammatory disorders such as multiple sclerosis and acute disseminated encephalomyelitis but also in epilepsies [19, 30, 31]. Childhood is the most common period for a variety of infections. Interestingly, the proinflammatory cytokines IFN-γ, IL-1β and IL-6 were negatively correlated with the age of disease onset in children with epilepsy, suggesting that younger patients demonstrated a more aggravated inflammatory response to recent seizure attacks. This childhood tendency toward hyper-responsiveness to acute seizure attacks may aggravate the proinflammatory response to diverse insults, such as infection, trauma and even febrile seizures, and may cause sustained neuroinflammation leading to epilepsy.

IL-1β is one of the most important proinflammatory cytokines produced in the CNS by activated microglia and astrocytes, as well as by macrophages and other immune cells in the periphery. IL-1β upregulates excitatory glutamatergic neurotransmission and lowers inhibitory gamma-aminobutyric acid (GABA)-mediated currents [32]. In experimental animals, IL-1β injection prolongs the duration of electroencephalographic seizure [33]. Epileptogenesis is a continuing process that begins before the first seizure and continues after epilepsy diagnosis. Considerable effort has been devoted to the search for biomarkers of epilepsy or ongoing epileptogenesis, including blood, brain imaging, electrophysiology and pathology [34, 35]. In traumatic brain injury, CSF/serum levels of IL-1β and IL-1β genetic variants were reported as prognostic factors for posttraumatic epilepsy risk in humans [21]. In our study, serum IL-1β levels within 48 h after afebrile seizure attacks showed correlation with the numbers of antiepileptic drugs being used by children with epilepsy. An increase in the number of antiepileptic drugs suggests drug resistance in patients, so the increase in serum IL-1β may correlate with drug resistance or with ongoing epileptogenesis or neuroinflammation. Interestingly, serum IL-1β did not correlate with the measure of disease severity in children with acquired demyelinating disorders of the CNS, the prototype of autoimmune neuroinflammation. Thus, serum IL-1β may be specific to neuroinflammation for epilepsy development, implicating it as a prognostic biomarker for epilepsy.

Expression of α-synuclein is increased during epileptogenesis [23, 36]. Status epilepticus triggers an increase of α-synuclein expression in the dentate gyrus of animal models with pilocarpine-induced epilepsy [23] and in the hippocampus of patients with mesial temporal lobe epilepsy [36]. In our study, serum α-synuclein was significantly increased in children with epilepsy and with acquired demyelinating disorders of the CNS, the prototype of autoimmune neuroinflammatory disorders of the CNS in childhood. Moreover, serum α-synuclein levels were positively correlated with measures of disease severity for both epilepsy and acquired disorders of the CNS, implying that serum α-synuclein is a potential prognostic biomarker of neurodegenerative processes. Serum levels of α-synuclein were also correlated with exosome α-synuclein levels, the presumptive fraction from the CSF. Moreover, exosomal α-synuclein levels showed significant correlation with serum IL-6 levels in children with epilepsy and acquired demyelinating disorders of the CNS. And the longer the duration of seizure, the higher serum IL-6 levels was in our children with epilepsy. In human astrocyte cell lines, α-synuclein can directly stimulate astrocyte production of IL-6 [37]. Astrocytes are a major source of proinflammatory cytokines, such as IL-1β and IL-6, even causing neurotoxicity. Therefore, correlation between the serum IL-6 levels and exosomal α-synuclein supports the viscous neurotoxicity cycle of neuroinflammation in epilepsy patients. Although exosome α-synuclein levels did not show a significant correlation with the measure of disease severity in patients with epilepsy and with acquired demyelinating disorders of the CNS in our study, probably due to the small number of patients available exosome fraction of serum. The sensitivity of the method used to extract exosomes from the peripheral blood could be problematic, and these commercially available methods are not free from protein contamination and may affect the properties of the exosomes [38].

In recent studies of children with autism spectrum disorder [39] and with attention-deficit/hyperactivity disorder, serum α-synuclein levels are reported to be low or no changes compared to controls [40]. Therefore serum α-synuclein levels may suggest neurodegenerative process related with neuroinflammation in children.

Our study has some limitations. First, a relatively small number of patients were enrolled, especially children with acquired demyelinating disorders of the CNS, as positive controls. Second, α-synuclein exists in several different forms, such as oligomeric, phosphorylated and total forms; however, we measured only the total form. Third, exosome α-synuclein should be measured by a more accurate method to increase sensitivity. Fourth, to distinguish neuroinflammation or neurodegeneration, such as neurocognitive dysfunction, neurocognitive tests for children with epilepsy at the time of initial and follow-ups must be conducted and compared with serum levels of α-synuclein and IL-1β.

## Conclusions

In summary, we demonstrated that serum levels of α-synuclein were significantly increased both in children with epilepsy and with acquired demyelinating disorders of the CNS and that serum α-synuclein was correlated with measures of disease severity. Serum levels of IL-1β showed a significant correlation with measures of disease severity only in children with epilepsy but not in those with acute demyelinating disorders of the CNS. Thus, these data support that serum α-synuclein are potential prognostic biomarkers for disease severity in children with epilepsy and acquired demyelinating disorders of the CNS and serum IL-1β is a specific biomarker for drug-resistance in children with epilepsy. However, further studies including larger sample sizes and neurocognitive testing are needed to confirm the usefulness of serum α-synuclein and IL-1β as prognostic biomarkers for epilepsy.

## Data Availability

The datasets used and/or analyzed during the current study are available from the corresponding author on reasonable request.

## Abbreviations

ADEM: acute disseminated encephalomyelitis
CNS: central nervous system
CSF: Cerebrospinal fluid
EEG: electroencephalogram
ELISA: enzyme-linked immunosorbent assay
GABA: gamma-aminobutyric acid
ICAM: Intercellular Adhesion Molecule
IFN: interferon
IL: interleukin
ILAE: International League Against Epilepsy
MRI: magnetic resonance imaging
NMOSD: neuromyelitis optica spectrum disorder
SD: standard deviation
TM: transverse myelitis
TNF: tumor necrosis factor
